# Behavioral Deficits Are Accompanied by Immunological and Neurochemical Changes in a Mouse Model for Neuropsychiatric Lupus (NP-SLE)

**DOI:** 10.3390/ijms160715150

**Published:** 2015-07-03

**Authors:** Yan Li, Amanda R. Eskelund, Hua Zhou, David P. Budac, Connie Sánchez, Maria Gulinello

**Affiliations:** 1 Lundbeck Research USA, Paramus, NJ 07652, USA; E-Mails: yli@Lundbeck.com (Y.L.); hzho@Lundbeck.com (H.Z.); dpbu@Lundbeck.com (D.P.B.); cs@lundbeck.com (C.S.); 2 Translational Neuropsychiatry Unit, Aarhus University, Risskov DK-8240, Denmark; E-Mail: ares@clin.au.dk; 3 Behavioral Core Facility, Department of Neuroscience, Albert Einstein College of Medicine, Bronx, NY 10461, USA

**Keywords:** MRL/lpr, lupus, forced swim test, anhedonia, novel object placement test, cytokines, chemokines, indoleamine-2,3-dioxygenase (IDO), kynurenine

## Abstract

Neuropsychiatric symptoms of systemic lupus erythematosus (NP-SLE) have been understudied compared to end-organ failure and peripheral pathology. Neuropsychiatric symptoms, particularly affective and cognitive indications, may be among the earliest manifestations of SLE. Among the potential pathophysiological mechanisms responsible for NP-SLE are increased peripheral pro-inflammatory cytokines, subsequent induction of indoleamine-2,3-dioxygenase (IDO) and activation of the kynurenine pathway. In the MRL/MpJ-*Fas^lpr^* (MRL/lpr) murine model of lupus, depression-like behavior and cognitive dysfunction is evident before significant levels of autoantibody titers and nephritis are present. We examined the behavioral profile of MRL/lpr mice and their congenic controls, a comprehensive plasma cytokine and chemokine profile, and brain levels of serotonin and kynurenine pathway metabolites. Consistent with previous studies, MRL/lpr mice had increased depression-like behavior and visuospatial memory impairment. Plasma levels of different inflammatory molecules (Haptoglobin, interleukin 10 (IL-10), interferon γ-inducible protein 10 (IP-10/CXCL10), lymphotactin, macrophage inhibitory protein 3β (MIP-3β/CCL19), monocyte chemotactic protein 1, 3 and 5 (MCP-1/CCL2, MCP-3/CCL7, MCP-5/CCL12), vascular cell adhesion molecule 1 (VCAM-1), lymphotactin and interferon γ (IFN-γ)) were increased in MRL/lpr mice. In cortex and hippocampus, MRL/lpr mice had increased levels of kynurenine pathway metabolites (kynurenine, 3-hydroxykynurenine, 3-hydroxynthranilic acid and quinolinic acid). Therefore, our study suggests that increased cytokine expression may be critical in the regulation subtle aspects of brain function in NP-SLE via induction of IDO and tryptophan/kynurenine metabolism.

## 1. Introduction

Systemic lupus erythematosus (SLE) is an autoimmune disease characterized by multiple abnormalities of the immune system, end organ pathology and a high incidence of neuropsychiatric symptoms [1,2], with roughly 40%–70% of SLE patients demonstrating cognitive and/or affective disorders [1]. Among the many ways that peripheral inflammatory mediators may directly alter brain function is loss of integrity of the blood-brain barrier, induction of specific inflammation in the brain, damage caused by brain-reactive autoantibodies and immune complexes, and altered tryptophan metabolism. Cytokines may increase indoleamine-2,3-dioxygenase (IDO) activity and produce kynurenine metabolites, kynurenic acid and quilnolinic acid, both of which affect glutamatergic transmission. Together with increased serotonin turnover or decreased serotonin levels, these mechanisms could potentially induce NP-SLE symptoms [3,4,5,6].

To date, neuropsychiatric and neurocognitive symptoms of systemic lupus erythematosus (NP-SLE) have been understudied compared to end-organ failure and peripheral pathology. Neuropsychiatric symptoms, particularly affective symptoms, may be among some of the earliest manifestations of SLE [2]. Approximately 40% of the NP-SLE symptoms develop before the onset of SLE or at the time of diagnosis and neuropsychiatric outcomes are evident independent from active disease states and end organ pathology [7,8]. This was found to be the case also in the animal model of lupus which is the subject of this study, the MRL/MpJ-*Fas^lpr^* (MRL/lpr) mouse, where depression-like behavior and cognitive dysfunction is evident in young animals before significant levels of autoantibody titers and kidney disease are evident [6,9].

MRL/lpr mice spontaneously develop hallmark diagnostic signs of SLE, including lymphoid hyperplasia, B cell hyperactivity, autoantibodies, circulating immune complexes, kidney disease, cognitive dysfunction and depression-like behavior in comparison to the congenic MRL+/+ control mice [6,9,10,11,12,13]. In MRL/lpr mice, depression-like behavior has been reported to correlate with the titer of autoantibodies [14]. However, recent evidence suggests that brain autoantibodies may not be as critical in neuropsychiatric lupus (NP-SLE) as previously thought as even complete deletion of B-cells does not reduce the phenotype [15,16]. In addition, MRL/lpr mice demonstrate elevated and aberrant cytokine expression [17,18,19,20,21,22]. However, despite the interest in the role of cytokines in regulating both peripheral and central aspects of lupus, often only a select subset of cytokines are examined when in reality there is a high degree of cross-talk and complex relationships between the various cytokine pathways and their downstream targets. Thus, we included a broad panel of cytokines and chemokines in our analysis.

**Figure 1:**
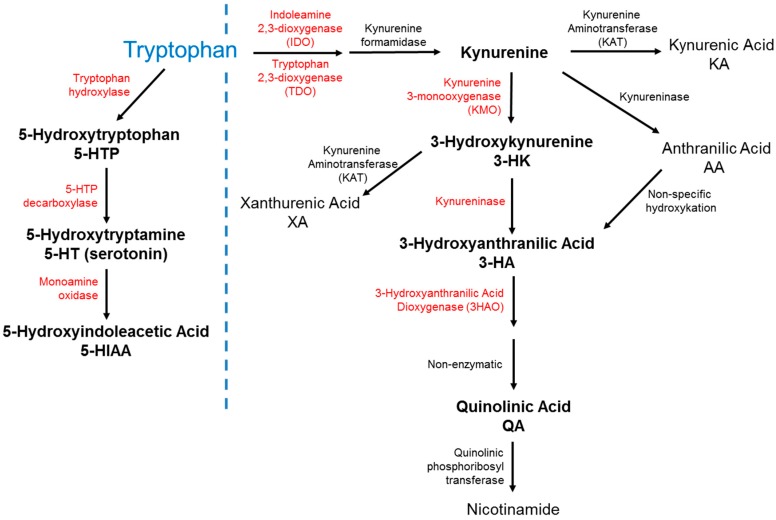
Major metabolites of tryptophan in serotonin and kynurenine pathways. Key enzymes are in red, and key metabolites indicated with bold font. The dotted line separates the tryptophan pathway from the IDO pathway.

One potential mechanism by which peripheral inflammatory signals might mediate NP-SLE could be through altered activity of indoleamine-2,3-dioxygenase (IDO, see Figure 1), an enzyme that is critical in shaping the inflammatory environment [23,24] and the neuroimmune interactions engendering NP-SLE [25]. IDO activity is increased in patients with lupus [26] and may be associated with altered neurotransmitter metabolism [27,28]. IDO activity is also regulated by inflammatory mediators in various models of acute and chronic inflammation [29,30] and can be associated with both cognitive deficits and affective dysfunction in response to inflammation [31,32,33,34,35,36,37,38], (reviewed in [39]). IDO catalyzes the conversion of tryptophan to kynurenine, which is associated with affective and cognitive symptoms [23,40,41] and may be important in the development of lupus [42,43,44]. Downstream metabolites of kynurenine include the *N*-Methyl-d-aspartate (NMDA) receptor antagonist, kynurenic acid [45], and the NMDA receptor agonist, hese neuroactive molecules in target brain regions can directly affect brain function. In additquinolinic acid [46]. Altering the balance between excitation and inhibition of synaptic transmission by tion, quinolinic acid may induce neuronal cell death [47], neuronal degeneration [48] and glial proliferation [49]. Increased quinolinic acid is related to affective and cognitive dysfunction [49,50,51,52,53,54], particularly in hippocampal dependent domains [55,56].

In this study, we characterize the behavioral profile of MRL/lpr mice (and their age matched congenic controls) and assess a comprehensive profile of cytokine and chemokine expression in addition to brain levels of tryptophan and serotonin metabolites reflecting IDO pathway activation.

## 2. Results

### 2.1. MRL/MpJ-Fas^lpr^ (MRL/lpr) Mice Exhibit Depression-Like Behavior without Anxiety-Like Behavior

Depression-like and anxiety-like behavior were measured between 10–15 weeks of age in MRL/lpr (LPR) mice and their congenic background strain MRL+/+ (MRL) mice.

**Figure 2:**
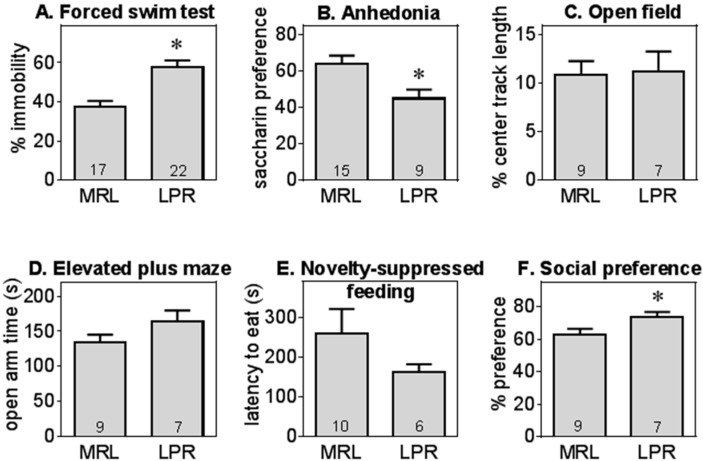
MRL/lpr mice displayed increased depression-like behavior without other changes in emotional domains. MRL/lpr (LPR) mice exhibited significantly higher immobility in forced swim test (Panel **A**) and anhedonia (Panel **B**); In contrast, anxiety-like behavior (center activity levels in open field Panel **C**; open arm time in elevated plus maze, Panel **D**; or latency to eat in the novelty-suppressed feeding test, Panel **E**) did not differ between LPR and MRL+/+ (MRL) mice overall, although LPR female mice exhibited a modest increase in social preference (social preference, Panel **F**). Data are shown as mean ± S.E., *, significant difference between LPR and MRL mice (*p* < 0.05 or *p* < 0.01). Sample sizes are shown within the bars for this and the following graphs.

**Figure 3:**
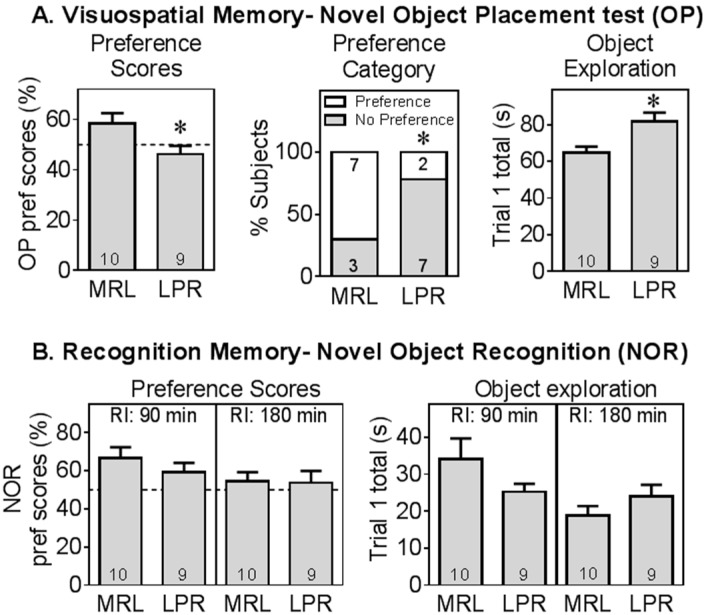
MRL/lpr mice displayed specific cognitive impairment. MRL/lpr (LPR) mice had deficits in visuospatial memory tested in the novel object placement test (Panel **A**), assessed as preference scores and as proportion had preference for moved (novel) object. LPR mice had higher object exploration than MRL+/+ (MRL) mice. However, LPR mice were not significantly different from MRL mice in the novel object recognition test (Panel **B**). The dotted line at 50% preference indicates chance performance *, significant difference between LPR and MRL mice (*p* < 0.05 or *p* < 0.01). RI: retention interval.

As shown in Figure 2A, MRL/lpr (LPR) mice exhibited increased depression-like behavior, assessed as immobility in forced swim test (*t* = 4.49, *df* = 37, *p* < 0.01), consistent with previous results [9,12]. Even though LPR mice had modestly lower locomotor activity (total tracklength in open field MRL: 2920 ± 130 cm, LPR: 2419 ± 97 cm), there was no significant correlation between the total tracklength and the immobility in forced swim test in either genotype and these activity levels were within normal ranges. Furthermore, exploration of novel objects, also an active process, was also normal in LPR mice (Figure 3).

LPR mice also displayed anhedonic behavior in the saccharin preference test (*t* = 2.64, *df* = 22, *p* < 0.05, Figure 2B), similar to previous reports [57] despite the equivalent total liquid consumption between groups (MRL: 0.95 ± 0.06 mL, LPR: 0.82 ± 0.10 mL). There was no difference in anxiety-like behavior between MRL and LPR mice, assessed as center activities in the open field test (Figure 2C), latency to feed in novelty suppressed of feeding (Figure 2D), or the amount of time spent in the open arms in elevated plus maze test (Figure 2E). In the social preference test, female LPR mice had a modestly higher preference for a conspecific (Figure 2F, *t* = 2.22, *df* = 14, *p* < 0.05).

### 2.2. MRL/lpr Mice Displayed Cognitive Deficits in Visuospatial Memory but Not in Object Recognition Memory

At 10 weeks of age, LPR mice exhibited visuospatial memory deficits in the object placement test (Figure 3A), assessed by preference scores (*t* = −2.34, *df* = 17, *p* < 0.05) or by success rate (χ^2^ = 4.54, likelihood ratio *p* < 0.05). This was not due to non-specific reduction in the response to novel objects, as during the training trial, LPR mice explored the objects more than MRL controls (*t* = 3.05, *df* = 17, *p* < 0.01). LPR mice did not have any deficit in recognition memory (novel object recognition test, Figure 3B). With a shorter retention interval of 90 min (RI), the difference between LPR and MRL mice was not significant. At a longer retention interval of 180 min, mice of both strains performed at random level (*i.e*., did not display a preference for the novel object). These results are consistent with previous reports [6].

### 2.3. MRL/lpr Mice Had Higher Levels of Several Plasma Biomarkers Related to Inflammation

**Figure 4:**
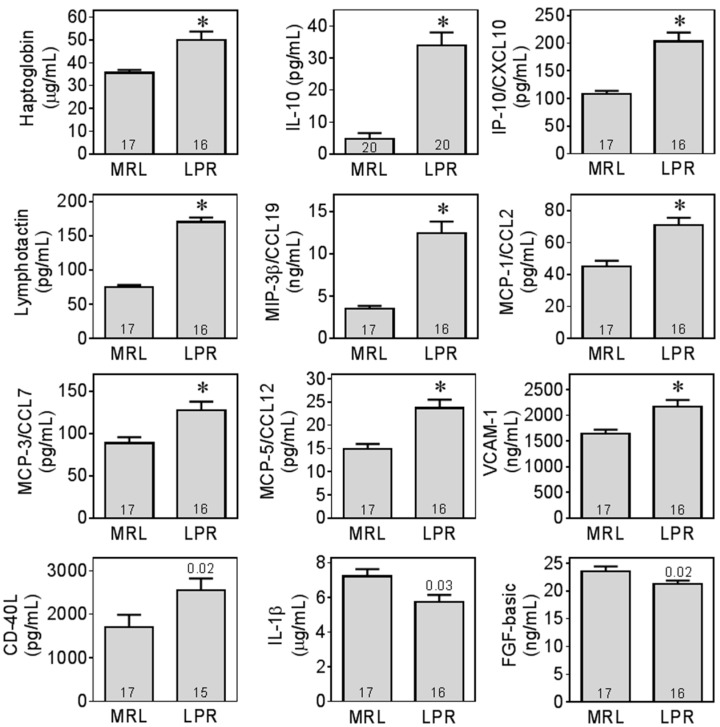
MRL/lpr mice had increased levels of inflammatory biomarkers in plasma. Biomarkers within detection limit in all groups are illustrated as absolute plasma concentrations. *, significant difference (*p* < 0.0026) between MRL/lpr (LPR) and MRL+/+ (MRL) after Bonferonni correction for multiple comparisons. For cytokines clearly elevated in LPR mice that did not reach this criterion of statistical significance, exact *p*-values were shown above LPR bars. For complete list of biomarkers and detailed statistical results, please refer to Table S1.

Multiple plasma biomarkers were screened in LPR and MRL mice (refer to Table S1 for a complete list). A subset of these biomarkers was within detection limits and exhibited differences between genotypes (Figure 4).

MRL/lpr (LPR) mice had higher plasma levels of biomarkers related to innate immune functions, such as macrophage inflammatory protein 3β (MIP-3β/CCL-19, *z* = 4.67, *p* < 0.0001), monocyte chemoattractant protein 1 (MCP-1/CCL-2, *z* = 3.66, *p* = 0.0003), monocyte chemoattractant protein 3 (MCP-3/CCL-7, *z* = 3.03, *p* = 0.0025), monocyte chemoattractant protein 5 (MCP-5/CCL-12, *z* = 3.31, *p* = 0.0009), and vascular cell adhesion molecule 1 (VCAM-1, *z* = 3.08, *p* = 0.0021). LPR mice also had higher levels of the biomarkers associated with adaptive immune system, such as interleukin 10 (IL-10) (*z* = 4.96, *p* < 0.0001) and interferon-γ inducible protein 10 (IP-10, *z* = 4.58, *p* < 0.0001). Furthermore, LPR mice had higher levels of lymphotactin (*z* = 4.88, *p* < 0.0001). In addition, haptoglobin (*z* = 3.77, *p* = 0.0002) was also increased in LPR mice.

**Figure 5:**
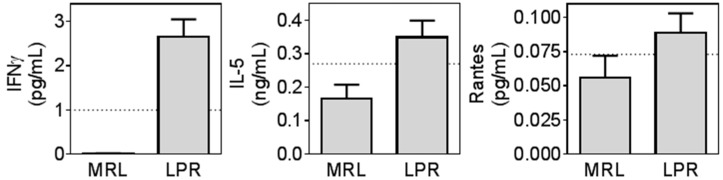
Biomarkers only detectable in MRL/lpr mice. Some cytokines were below detection limits in many of the MRL mice but detectable in LPR mice. Undetectable values were assigned a as 0 value and illustrated (*n* = 16–20 in each group) but not analyzed statistically. Dotted lines: detection limit.

A few biomarkers (interferon γ (IFNγ), interleukin 5 (IL-5) and Rantes) were undetectable in all or most MRL mice, however were detectable in all or most LPR mice. Missing values prevented full statistical analysis on these biomarkers but are illustrated in Figure 5.

### 2.4. MRL/lpr Mice Exhibit Altered Tryptophan and Kynurenine Metabolism

**Figure 6:**
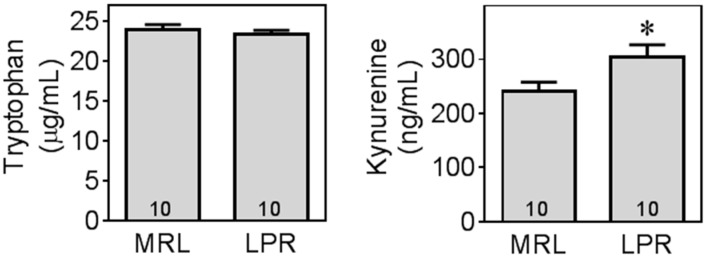
MRL/lpr mice had higher levels of kynurenine in plasma. Plasma levels of tryptophan (TYP) and kynurenine (KYN) were measured using HPLC. *, significant difference between LPR and MRL mice at *p* < 0.05.

Although there was no difference in plasma tryptophan levels between MRL and LPR mice (Figure 6), LPR mice had a significantly higher level of kynurenine (*t* = 2.32, *df* = 18, *p* < 0.05), which also resulted in a significantly higher kynurenine to tryptophan ratio (*MRL* = 0.0101 ± 0.0007, *LPR* = 0.0132 ± 0.0011, *t* = 2.29, *df* = 18, *p* < 0.05).

**Figure 7:**
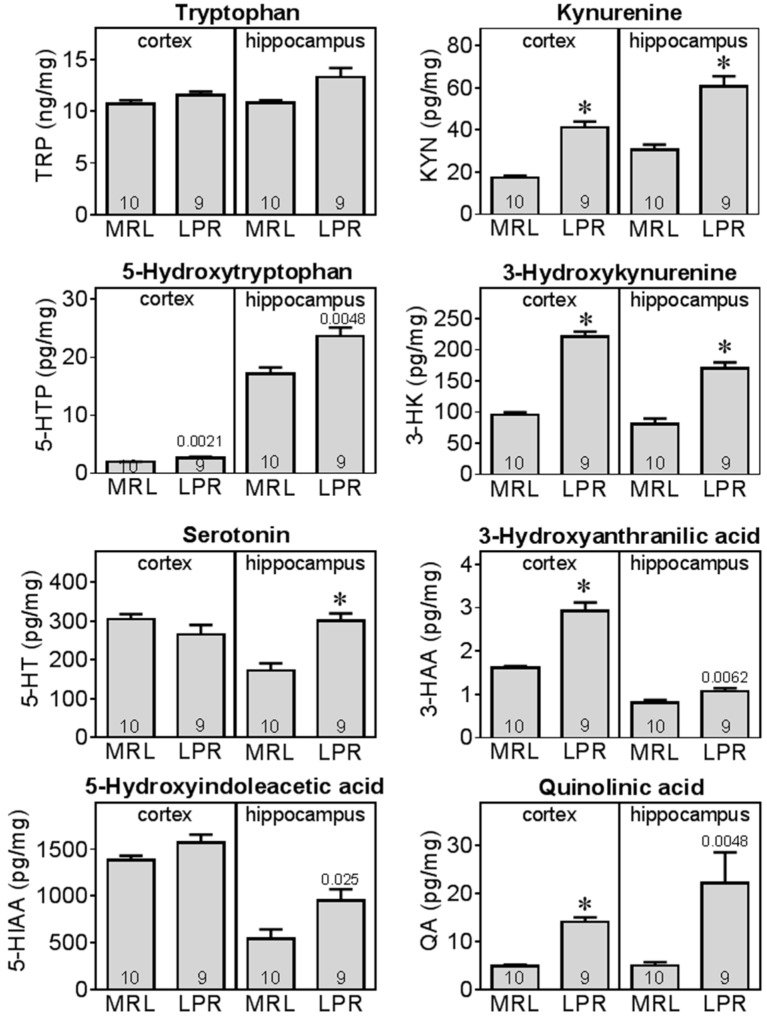
MRL/lpr mice had altered tryptophan metabolism in frontal cortex and hippocampus. Various metabolites of tryptophan were measured in brain tissue homogenates using HPLC methods. *, significant difference (*p* < 0.0021) between MRL/lpr (LPR) and MRL+/+ (MRL). For metabolites elevated in LPR mice but which did not reach this criterion of statistical significance, specific *p*-values are shown. For a complete list of metabolites and detailed statistical results, please refer to supplementary Table S2.

Levels of tryptophan metabolites were further measured in both frontal cortex and hippocampus. Similar to the results in plasma, there was no significant difference in tryptophan levels in brain (cortex and hippocampus), but LPR mice had altered tryptophan metabolism along both kynurenine and serotonin pathways in these brain regions (Figure 7).

There was a significant increase in kynurenine pathway metabolism in LPR mice. In cortex, kynurenine (KYN), 3-hydroxykynurenine (3-HK), 3-hydroxyanthranilic acid (3-HAA), and quinolinic acid (QA) were significantly (*p* < 0.0021) higher in LPR mice compared to MRL. In hippocampus, KYN, 3-HK levels were also significantly higher than MRL, with increases in 3-HAA and QA close to significance.

Increase in kynurenine resulted in a significant higher kynurenine/tryptophan (KYN/TRP) ratio in LPR mice cortex (MRL 0.0017 ± 0.0002; LPR 0.0036 ± 0.0003) and hippocampus (MRL 0.0028 ± 0.0002; LPR 0.0046 ± 0.0003). Increase in quinolinic acid resulted in a significantly higher quinolinic acid/tryptophan (QA/TRP) ratio (MRL 0.00046 ± 0.00002; LPR 0.00124 ± 0.0001), and quinolinic acid/kynureninc acid (QA/KA) ratio (MRL 11.4 ± 1.3, LPR 26.8 ± 1.5) in LPR mice cortex. Increases in QA/TRP (*p* = 0.008) and QA/KA (*p* = 0.01) ratios were also near significance in hippocampus. These results indicate increases in kynurenine pathway enzyme activities, including that of indoleamine-2,3-dioxygenase (IDO).

The serotonin pathway was also altered in the hippocampus of LPR mice. Levels of 5-hydroxytryptophan, serotonin and 5-hydroxyindoleacetic acid were significantly or near significantly increased compared to MRL mice. These results indicate increases in serotonin turnover in LPR mice, more profoundly in the hippocampal region.

## 3. Discussion

Behavioral tests in the murine MRL/MpJ*-Fas^lpr^* (MRL/lpr or LPR) model of neuropsychiatric lupus (NP-SLE) confirmed cognitive deficits and depression-like behavior, concomitant with increased levels of specific cytokines and altered brain metabolites of the indoleamine-2,3-dioxygenase (IDO) pathway, although these were not associated with decreased brain levels of tryptophan or serotonin.

Lupus-induced changes in chemokines and cytokines were consistent with both preclinical and clinical findings and indicate specific pathways that might be involved in the development and severity of lupus and NP-SLE symptoms [58], including increased expression of IFNγ [59,60,61,62], IL-10 [62,63,64,65,66,67], IP-10/CXCL10 [68,69,70,71,72,73,74], and MCP-1/CCL2 [71,72,73,74,75,76,77]. These results also identified other markers that are less well studied, but are also likely to be related to NP-SLE including VCAM-1 [71,75,78,79], haptoglobin [80], and MIP-3β/CCL-19 [74]. In addition, plasma levels of MCP-3/CCL7, MCP-5/CCL12, and lymphotactin were increased in the current study, for which we have found no prior reports in animal models or in patients. These mediators are associated with both innate (MCP-1, MCP-3, MCP-5, MIP-3β), adaptive immune systems (lymphotactin, IL-10, IP-10, IFNγ, IgA), or mediate leukocyte-endothelial cell interaction (VCAM-1), regulating diverse immune cells such as granulocytes, monocytes, dendritic cells, T- and B- lymphocytes. Interestingly, some inflammatory mediators had lower levels in MRL/lpr mice, including IL-1β and basic FGF. IL-1β is generally produced by activated macrophages and dendritic cells in response to alarmins. Thus, although IL-1β typically increases in response to pathogens and tissue damage, it does not appear to be involved in NP-SLE at this stage of the disease and it may be that the danger associated molecules (endogenous molecules released from dead cells) are not yet present in sufficient quantities to activate its secretion. Basic fibroblast growth factor is present at high levels in blood vessels and is increased at the site of wounds, so it is tempting to speculate that lower levels of FGF in lupus mice may represent a deficit of vascular growth or repair mechanisms [81,82,83,84,85]. There are some cytokines which were notably not elevated in this study in MRL/lpr mice (such as IL-6 and TNFα) although these were not present at detectable levels in our assay. The fact that we investigated a relatively early time point may be one reason for this discrepancy.

These inflammatory mediators may have negative effects on brain functions via different mechanisms. Firstly, cytokines can induce the indoleamine-2,3-dioxygenase (IDO) activity and produce neurotoxic metabolites. It has been well documented that IFNγ induces IDO expression and activity [39,41,86,87,88]. Other cytokines, such as IL-10 [89,90], IL-5 [91] and CD40-L [92] have also been shown to cooperate with IFNγ to induce IDO. Increased IDO activity can suppresses inflammatory response [93], which may be a homeostatic reaction. However, elevation of the kynurenine path of tryptophan metabolism has been linked to neuropsychological effects such as depression [38,88] and cognitive impairment [43,94,95]. Furthermore, soluble inflammatory mediators and other agents can compromise the integrity of the blood-brain barrier. VCAM-1 [96], MCP-1/CCL2 [97], IP-10 [98] can all increase the permeability of the blood-brain barrier and allow leukocytes to enter the brain and propagate neuroinflammation. Indeed, MCP-1/CCL2 has been used as a biomarker for neurological diseases associated with infection [99], and correlates to faster cognitive decline in prodromal Alzheimer’s disease [100]. These studies suggest that inflammatory mediators increased in MRL/lpr mice can negatively impact neuropsychiatric functions by a variety of mechanisms.

The alterations in the IDO pathway are themselves intrinsically linked to neuropsychiatric symptoms. Several metabolites in this pathway are neuroactive. Quinolinic acid is an NMDA receptor agonist [54] that causes excitotoxic effects *in vitro* [101] and *in vivo* [54]. 3-hydroxykynurenine (3-HK) generates reactive radical species, inducing oxidative stress and apoptosis in neurons [102,103]. In contrast, kynurenic acid is a noncompetitive NMDA receptor antagonist [45], which may counteract the toxic effect of quinolinic acid. However, in the current study, there was increase in quinolinic acid but not in kynurenic acid, resulting in an increase of QA/KA ratio in MRL/lpr mice, indicating the balance tends towards excitotoxicity. Increases in the kynurenine pathway are identified as mediators for inflammation-induced conditions such as depression and cognitive impairment [23,43,104]. Increased IDO activity has been shown to mediate inflammation-induced depression-like behavior in rodents [33,34,35] and in patients [88]. Furthermore, increased kynurenine after IFNα treatment is associated with depression onset in patients [105]. Quinolinic acid has been shown to influence memory impairment [49,51], is associated with depression in patients [53] and associated with NP-SLE [106]. Therefore, our findings are consistent with current theories that increased metabolites along the kynurenine pathway underlie cognitive and affective impairments associated with peripheral inflammation and may provide a link between peripheral inflammation and local CNS dysfunction. Metabolites downstream of indoleamine-2,3-dioxygenase (IDO) might be therapeutic candidates for alleviating NP-SLE symptoms.

It is worth noting that even at this relatively early stage of the disease, hippocampal function is affected in MRL/lpr mice. MRL/lpr mice exhibited deficits in object placement test for visuospatial memory, a task considered to be hippocampal dependent [107], but retained intact recognition memory [108,109]. This is consistent with previous findings in LPR mice [6] and other models of neuroinflammation-induced cognitive impairment [110]. In the current study, we also observed more profound tryptophan metabolism changes in hippocampal region compared to frontal cortex, with indications of increased serotonin turnover and kynurenine metabolite production. Therefore, brain neurochemical alterations in LPR mice are consistent with the behavioral outcomes we report.

## 4. Materials and Methods

### 4.1. Animals and Materials

MRL+/+ (MRL) and MRL/lpr (MRL/MpJ-*Fas^lpr^*, LPR) were purchased from Jackson Labs (Bar Harbor, ME, USA). Female mice were used in this study unless otherwise stated. Animals were group housed (three per cage) under 12:12 light: dark cycle (lights on 6 am, lights off 6 pm), in controlled environment of 20 °C and 60% humidity. Mice had *ad libitum* access to water and food, and study conducted when animals were 10–15 weeks of age. In previous studies, behavioral phenotypes were stable in this age range [6,9,11]. All experiments were conducted in accordance to the guidelines of NIH and AAALAC and approved by Lundbeck Research USA Institutional Animal Care and Use Committee.

### 4.2. Behavioral Tests

Animals were brought into the laboratory for acclimatization at least 30 min prior to behavioral testing. All tests were conducted under low levels of incandescent lighting between 9 am and 5 pm.

Open field test: Animals were allowed to freely explore a testing arena (50 cm × 50 cm × 35 cm) for 6 min and their activities were analyzed using tracking software (Viewer, Biobserve, Bonn, Germany). General locomotor activity was assessed as total track length and anxiety-like behavior as the proportion of activity occurring in the center of the arena (% center activity = 100 × center track length/total track length).

Object placement test for visuospatial memory: Visuospatial memory was examined using a novel object placement test (also known as novel object location test, place recognition test, spatial novelty test) [6,107]. Briefly, mice were first allowed to explore in an open field containing two identical objects (with high contrast intra-arena visual cues) for 5 min (Trial 1—Training Trial). The amount of object exploration (defined as rearing on, whisking, sniffing or touching the objects with nose and/or forepaws) was scored manually using stopwatches. After a retention interval of 20 min, mice were returned to the same testing arena for another 3 min (Trial 2—Testing Trial), with one object moved to a different location. Exploration of each object was again manually scored. Results of the object placement test were reported as novel object placement preference scores (100% × exploration of relocated object during testing trial/total exploration during testing trial). Animals with intact visuospatial memory preferentially explore the relocated (novel) object and thus would have a preference score >50%. Total novel object exploration (s) during Trial 1 is also illustrated as an internal control. Track length was measured by Viewer tracking software (Biobserve, Bonn, Germany). Results were also reported as success rates—the proportion of animals in each group performing higher than chance (*i.e*., preferring the novel object). For this purpose, preference scores higher than 55 were defined as “passing”, based on the following rationale: during the initial and extensive validation of these tasks in Einstein Behavioral Core Facility, it was determined that animals with preference scores higher than 55 consistently demonstrate novel object preferences when retested few animals had scores between 53 and 55; and the use of the less strict criterion (53) makes little difference to the analysis and has been previously validated and published [111,112,113,114,115].

Novel object recognition test for recognition memory: Recognition memory was assessed using the novel object recognition test [6,116]. Briefly, mice were allowed to explore two identical objects in the testing arena for 3 min (Trial 1—Training Trial). After a retention interval of 3 h, mice were returned to the arena for another 3 min, with one of the familiar objects now replaced by a novel object (Trial 2—Testing trial). The amount of exploration was scored manually. As in the object placement test, the performance was evaluated by preference score (100% × exploration during testing trial novel object/total testing trial exploration).

Novelty suppression of feeding: Animals were tested for anxiety-like behavior in a modified novelty suppressed feeding test [117]. Mice were food deprived overnight before testing (with *ad libitum* access to drinking water). During the test, animals were released from corner into a brightly lit testing box (50 cm × 50 cm), in the center of which food pellets were placed. Latency to begin eating (amount of time it took for the animal to start eating food pellet in the center of arena) was recorded manually with stop watch. If an animal did not start eating within 10 min, the test was terminated and a score of 600 sec was assigned to the animal. Animals were assessed to ensure they consumed food in their home cages following the test.

Elevated plus maze: Animals were allowed to explore an elevated plus maze (Lafayette Instrument Company, Lafayette, CA, USA) with 2 open arms and 2 closed arms (arm length: 35 cm, arm width: 5 cm, wall height: 15 cm, leg height: 40 cm) for 10 min. The period of animal spent in exploring the open arm was scored using a stop watch manually, with great time spent in the open arms indicating lower levels of anxiety-like behavior [118].

Social preference: Animals were placed in the start arm of a 3-chambered Y-maze and allowed to choose freely between two baited arms that contain either an inanimate object or an ovariectomized female conspecific stimulus animal, both behind Plexiglas barriers. The amount of exploration (defined as sniffing, rearing, whisking, orientation and physical contact) of each area was recorded manually for 5 min. Results are expressed as social preference scores: 100 × (exploration of stimulus animal/total exploration). General locomotor activity was assessed as tracklength using Viewer automated tracking software (Biobserve, Bonn, Germany).

Two bottle choice test for anhedonia: Lack of response to reward or pleasure (anhedonia) was tested using a two bottle choice test for saccharin preference. During the 2-day pre-exposure period, animals were exposed to two drinking bottles containing either tap water or saccharin sweetened tap water. Saccharin concentrations were increased from the first night (0.025%) to the second night (0.1%). Rats were then water deprived on the third night. On the fourth morning, animals were individually caged, allowed to acclimatize to the cage for 30 min and then provided with water and 0.5% saccharin in a standard 2-bottle choice test. Both bottles were weighed at 30 min, 1 h, 90 min, and 2 h during the test. Positions of the drinking bottles were counter balanced and switched after each weighing during testing and during pre-exposure. Results were expressed as saccharin preference scores: 100 × (saccharin intake/total liquid intake). Anhedonia was defined as a reduction in saccharin preference relative to control subjects.

Forced swim test: Animals were tested for depression-like behavior in a modified forced swim test [119,120]. Mice were placed in Plexiglas cylinders (diameter 25 cm) containing 27 °C water (30 cm deep) for 7 min. Duration of immobility (defined as no movement other than what is necessary to keep head above water) was scored manually using stop watches, excluding the first minute of the trial. At the end of test, mice were dried in warming cages with a thermal gradient created by heating pad and heating lamp for 15 min, before returned to their home cages. Water was changed between animals. Results were expressed as percentage of trial period that animal was immobile (%immobility).

### 4.3. Sample Collection

Animals were euthanized by carbon dioxide inhalation followed by decapitation. Trunk blood was collected into EDTA containing tube (BD Diagnostics, Franklin Lakes, NJ, USA). Plasma was separated by centrifugation (3000 rpm, 15 min at 4 °C) and stored in −80 °C until further analysis. Frontal cortex and hippocampus were dissected, snap-freeze and stored in −80 °C until analysis.

### 4.4. Quantification of Chemokines and Cytokines

Chemokines, cytokines and other biomarkers were measured in plasma samples by Rules Based Medicine (Austin, TX, USA), using a customized Luminex^®^-based Multi-Analyte Profile (MAP) technology platform (Luminex, Austin, TX, USA). Furthermore, a subset of cytokines were analyzed separately using customized multiplex assay based on electrochemiluminescence technology purchased from Meso Scale Discovery (Rockville, MD, USA). Manufacture’s assay instructions were strictly followed. We pre-defined inflammatory biomarkers for analysis based on relevant disease biology. Some biomarkers were only detectable in LPR mice but not in the majority of MRL mice, therefore statistical analysis could not be performed. These were illustrated in a separate figure, with 0 assigned to the undetectable values in the MRL mice.

### 4.5. Liquid Chromatography-Tandem Mass Spectrometry Analysis

Standard curves were prepared using pure components (tryptophan (TRP), kynurenine (KYN), kynurenic acid (KYNA), 3-hydroxykynurenine (3HK), xanthurenic acid (XT), quinolinic acid (QA), nicotinamide (NTA), anthranilic acid (AA), 3-hydroxyanthranilic acid (3HAA), picolinic acid (PA), serotonin (5HT), kynuramine (KYNAM) 5-hydroxytryptophan (5HTP), 5-hydroxyindole acetic acid (5HIAA)) purchased from Sigma Aldrich (St. Louis, MO, USA) dissolved in 0.2% acetic acid. 5-hydroxykynurenine was prepared internally and prepared in the same manner. Internal standards were obtained from Sigma Aldrich (^13^C_11_, ^15^N_2_-TRP, ^2^H_5_-KYNA, ^2^H_4_-NTA, ^2^H_4_-PA, ^2^H_4_-5HT) or Alsachim (Illkirch Graffenstaden, France: ^13^C_6_-3HK, ^13^C_6_-KYN, ^13^C_6_-3HAA, ^13^C_4_, ^15^N-QA) and added to each standard and sample for a final concentration of 50 ng/mL to correct for sample and instrument variability.

Plasma or tissue blocks for homogenization were diluted 5 fold (*w*/*v*) by adding 0.2% acetic acid aqueous solution containing internal standards. Frontal cortical and hippocampal samples were homogenized using AFA™ Fiber tubes with cap (Covaris, Woburn, MA, USA) on a FastPrep^®^-96 Instrument (MP Biomedicals, LLC., Santa Ana, CA, USA) for 5 min at 1200 oscillations/min. Diluted samples were then filtrated through 3 kDa Amicon Ultra filter (Millipore, Billerica, MA, USA) by centrifugation at 13,500× *g* for 60 min at 4 °C. Resultant solutions were directly injected into a Waters (Milford, MA, USA). Acquity HPLC system equipped with an YMC™ ODS-AQ™ 2 mm × 100 mm, 3 μm particle column. Separated kynurenine analytes were detected by a Waters Quattro Premier XE triple quadrupole mass spectrometer (Waters, Milford, MA, USA), operating in the MS/MS mode. Column and pre-column tubing were maintained at 40 °C while eluting kynurenine metabolites with a mobile phase consisting of an aqueous component (A: 0.5% formic acid in milliQ water) and an organic component (B: 1% formic acid in acetonitrile). Gradient elution included a 2 min hold at 100% A followed by a shallow gradient of 0%–30% B over 4.4 min. Later eluting materials were then brought off the column using a stronger gradient of 30%–50% B over 0.5 min with a total run time of 9 min. Quantifications of analytes were determined by comparing to the internal standards of known concentrations and averaged on triplicate determinations. Limits of detection were determined based on a signal to noise ratio of >10 and found to be well below required limits for analysis of these analytes in rodent samples.

### 4.6. Statistical Analysis

Data are shown as mean ± SEM with sample sizes indicated within the bars in each figures. Differences between LPR and MRL mice were analyzed using student’s *t*-test for preference scores in memory tests, plasma tryptophan and kynurenine levels, and chi-square test was used for compare success rates in memory test. Significance was defined as *p* < 0.05. Plasma cytokines and brain tryptophan metabolites were analyzed using Wilcoxon/Kruskal-Wallis Rank Sums test (calculates *z*-values, a nonparametric equivalent for *t*-test) with Bonferroni correction for multiple comparisons (cytokines: *p* < 0.0026; tryptophan metabolites: *p* < 0.0021). All statistical tests were performed using JMP10 software (SAS, Cary, NC, USA).

## 5. Conclusions

MRL/lpr mice displayed depression-like behavior and cognitive impairment at a time before systemic disease manifestation [9]. These behavioral changes were accompanied by increased plasma levels of inflammatory mediators and increased brain levels of kynurenine metabolites. This study indicates that in this murine model for NP-SLE, peripheral inflammation is accompanied by NP-SLE symptoms and increased kynurenine metabolites are concomitant with elevated levels of numerous plasma cytokines.

## Supplementary Materials

Supplementary materials can be found at http://www.mdpi.com/1422-0067/16/07/15150/s1.
