# Supplementary material for: Behavioral Deficits Are Accompanied by Immunological and Neurochemical Changes in a Mouse Model for Neuropsychiatric Lupus (NP-SLE)
Source: Int J Mol Sci. 2015 Jul 3;16(7):15150–71. doi: 10.3390/ijms160715150 (PMC4519892; doi:10.3390/ijms160715150)
Supplement: Supplementary file 1 [file ijms-16-15150-s001.pdf]

Supplementary Information

Table S1. Results of plasma biomarker screening.

Biomarker	Unit	Detection Limit	MRL	LPR	Significance
Apo A-I	µg/mL	1.4	25.7 ± 1.1	27.3 ± 1.0	n.s.
CD40	pg/mL	5.3	70.6 ± 6.7	67.2 ± 7.7	n.s.
**CD40-L**	**pg/mL**	**508**	**1702.9 ± 286.8**	**2552.7 ± 288.9**	***p* = 0.02**
C-reactive protein (CRP)	µg/mL	0.86	4.9 ± 0.2	4.9 ± 0.2	n.s.
Eotaxin	pg/mL	5	620.6 ± 34.8	728.4 ± 60.0	n.s.
Epidermal growth factor (EGF)	pg/mL	9.3	681.5 ± 195.5	403.0 ± 140.4	n.s.
Factor VII	ng/mL	28	42.3 ± 0.8	41.6 ± 0.7	n.s.
Fibrinogen	mg/mL	0.178	59.1 ± 4.3	65.0 ± 3.6	n.s.
**Fibroblast growth factor-basic (FGF-basic)**	**ng/mL**	**23**	**23.6 ± 0.9**	**21.3 ± 0.6**	***p* = 0.02**
Granulocyte chemotactic protein-2 (GCP-2)	ng/mL	0.38	4.2 ± 0.4	4.2 ± 0.3	n.s.
**Haptoglobin**	**µg/mL**	**3.6**	**35.5 ± 1.4**	**50.0 ± 3.7**	*
Immunoglobin A (IgA)	µg/mL	3.7	609.5 ± 47.2	3620 ± 502.5	*
**Interferon-γ**	**pg/mL**	**1**	**u.d.**	**2.6**	**n.a.**
**Interferon-γ inducible protein 10 (IP-10)**	**pg/mL**	**5.1**	**107.7 ± 6.2**	**202.9 ± 16.3**	*
IL-1α	pg/mL	30	864.0 ± 206.1	499.1 ± 145.3	n.s.
**IL-1β**	**ng/mL**	**6**	**7.2 ± 0.4**	**5.7 ± 0.4**	***p* = 0.03**
**IL-5**	**ng/mL**	**0.41**	**u.d.**	**0.35 ± 0.05**	**n.a.**
IL-7	ng/mL	0.13	0.14 ± 0.02	0.13 ± 0.02	n.s.
**IL-10**	**pg/mL**	**1**	**4.7 ± 1.9**	**34.0 ± 4.0**	*
IL-18	ng/mL	4.3	20.4 ± 0.9	20.0 ± 0.5	n.s.
**Leukemia inhibitory factor (LIF)**	**pg/mL**	**707**	**1820.0 ± 48.2**	**1974.8 ± 45.8**	***p* = 0.02**
**Lymphotactin**	**pg/mL**	**9.9**	**74.7 ± 3.5**	**169.9 ± 6.7**	*
Macrophage colony-stimulating factor-1 (M-CSF-1)	ng/mL	0.0088	5.2 ± 0.2	4.7±0.1	n.s.
**Macrophage inflammatory protein-1α (MIP-1α)**	**ng/mL**	**2.9**	**7.6 ± 0.3**	**6.6 ± 0.2**	***p* = 0.006**
**Macrophage inflammatory protein-1β (MIP-1β)**	**pg/mL**	**29**	**168.2 ± 13.1**	**206.9 ± 9.2**	***p* = 0.01**
**Macrophage inflammatory protein-1γ (MIP-1γ)**	**ng/mL**	**0.92**	**36.8 ± 1.8**	**30.6 ± 2.0**	***p* = 0.03**
**Macrophage inflammatory protein-2 (MIP-2)**	**pg/mL**	**6**	**6.8 ± 0.9**	**4.5 ± 0.4**	***p* = 0.04**
**Macrophage inflammatory protein-3β (MIP-3β)**	**ng/mL**	**0.22**	**3.5 ± 0.3**	**12.4 ± 1.4**	*

Table S1. *Cont.*

Biomarker	Unit	Detection limit	MRL	LPR	Significance
Macrophage-derived chemokine (MDC)	pg/mL	44	902.8 ± 56.3	1077.2 ± 81.5	n.s.
Matrix metalloproteinase-9 (MMP-9)	ng/mL	7.8	52.8 ± 5.4	49.9 ± 4.8	n.s.
**Monocyte chemotactic protein-1 (MCP-1)**	**pg/mL**	**3.8**	**45.0 ± 3.7**	**70.9 ± 4.7**	*
**Monocyte chemotactic protein-3 (MCP-3)**	**pg/mL**	**5.2**	**88.5 ± 7.2**	**127.4 ± 10.2**	*
**Monocyte chemotactic protein-5 (MCP-5)**	**pg/mL**	**2.3**	**14.9 ± 1.1**	**23.7 ± 1.9**	*
Myeloperoxidase (MPO)	ng/mL	0.61	151.4 ± 14.8	166.5 ± 13.2	n.s.
**Myoglobin**	**ng/mL**	**6.8**	**1217.8 ± 276.5**	**286.9 ± 47.3**	*
Serum amyliod P-component (SAP)	µg/mL	2.3	27.4 ± 1.3	29.0 ± 1.3	n.s.
**Serum glutamic oxaloacetic transaminase (SGOT)**	**µg/mL**	**53**	**1478.2 ± 188.7**	**318.9 ± 19.9**	*
Stem cell factor (SCF)	pg/mL	19	220.6 ± 26.9	211.2 ± 21.1	n.s.
**RANTES**	**pg/mL**	**0.073**	**u.d.**	**0.089 ± 0.014**	**n.a.**
Thrombopoietin (TPO)	ng/mL	22	34.8 ± 1.3	33.9 ± 2.3	n.s.
Tissue factor (TF)	ng/mL	3.6	6.1 ± 0.4	6.0 ± 0.3	n.s.
**Tissue inhibitor of metalloproteinases 1 (TIMP-1)**	**ng/mL**	**0.041**	**0.8 ± 0.0**	**1.0 ± 0.1**	$p = 0.03$
**Vascular cell adhesion molecule-1 (VCAM-1)**	**ng/mL**	**2.8**	**1643.5 ± 78.0**	**2163.8 ± 136.2**	*
Vascular endothelial growth factor A (VEGF-A)	pg/mL	24	8823.2 ± 4093.2	7345.0 ± 3384.7	n.s.
von Willebrand factor (vWF)	ng/mL	15	103.7 ± 19.7	59.5 ± 6.0	n.s.

Plasma biomarkers were measured using multiplex assays. n = 16–20 per group. Wilcoxon Test (non-parametric equivalent of *t*-test) with Bonferroni correction was used to compare MRL+/+ (MRL, control) and MRL/lpr (LPR, lupus) mice. Biomarkers with significant (*, $p < 0.0026$) or substantially elevated differences (exact p value) between LPR and MRL are highlighted using bold font. u.d.: undetectable; n.s.: not significant; n.a.: cannot be calculated.

Table S2. Results of tryptophan metabolite analysis.

Metabolite	Unit	Frontal Cortex			Hippocampus		
		MRL	LPR	Significance	MRL	LPR	Significance
Tryptophan (TRP)	ng/mg	10.69 ± 0.38	11.53 ± 0.35	n.s.	10.83 ± 0.23	13.28 ± 0.89	n.s.
**5-Hydroxytryptophan (5-HTP)**	**pg/mg**	**1.97 ± 0.07**	**2.66 ± 0.21**	***p* = 0.0021**	**17.16 ± 1.11**	**23.63 ± 1.52**	***p* = 0.0048**
**5-Hydroxytryptamine (5-HT, serotonin)**	**pg/mg**	304.65 ± 42.94	265.31 ± 74.69	n.s.	**172.02 ± 61.83**	**301.43 ± 55.20**	*
**5-Hydroxyindoleacetic acid (5-HIAA)**	**pg/mg**	1383.49 ± 49.00	1569.44 ± 88.74	n.s.	**540.80 ± 101.32**	**950.86 ± 121.30**	***p* = 0.025**
**Kynurenine (KYN)**	**pg/mg**	**17.47 ± 0.82**	**41.27 ± 2.80**	*	**30.61 ± 2.40**	**60.69 ± 4.75**	*
**Kynurenic acid (KYNA)**	**pg/mg**	**0.44 ± 0.02**	**0.53 ± 0.02**	***p* = 0.008**	0.67 ± 0.06	0.86 ± 0.1	n.s.
Anthranilic acid (AA)	pg/mg	0.32 ± 0.03	0.41 ± 0.02	n.s.	0.63 ± 0.08	0.61 ± 0.06	n.s.
**3-Hydroxykynurenine (3-HK)**	**pg/mg**	**95.77 ± 3.99**	**221.37 ± 7.77**	*	**80.66 ± 8.98**	**170.07 ± 9.78**	*
Xanthurenic acid (XA)	pg/mg	0.44 ± 0.02	0.46 ± 0.01	n.s.	1.10 ± 0.06	1.26 ± 0.05	n.s.
**3-Hydroxyanthranilic acid (3-HAA)**	**pg/mg**	**1.61 ± 0.04**	**2.92 ± 0.20**	*	**0.81 ± 0.06**	**1.07 ± 0.07**	***p* = 0.0062**
**Quinolinic acid (QA)**	**pg/mg**	**4.87 ± 0.27**	**14.09 ± 0.93**	*	**4.93 ± 0.75**	**22.18 ± 6.47**	***p* = 0.0048**
Nicotinamide (NTA)	ng/mg	64.32 ± 0.86	64.37 ± 1.62	n.s.	46.11 ± 1.13	49.28 ± 1.58	n.s.

Tryptophan metabolites in brain homogenate derived from frontal cortex or hippocampus were measured using HPLC (n = 9–10 per group). Wilcoxon test with Bonferroni correction was used for statistical analysis. Metabolites with significant difference (*, $p < 0.0021$) or close significant difference (exact p-values shown) between MRL/lpr (LPR, lupus) and MRL+/+ (MRL, control) mice are highlighted using bold font. n.s.: not significant.
